# Effect of feeding regimen on meat quality, MyHC isoforms, AMPK, and PGC‐1α genes expression in the biceps femoris muscle of Mongolia sheep

**DOI:** 10.1002/fsn3.1494

**Published:** 2020-04-14

**Authors:** Yanru Hou, Lin Su, Rina Su, Yulong Luo, Bohui Wang, Duo Yao, Lihua Zhao, Ye Jin

**Affiliations:** ^1^ College of Food Science and Engineering Inner Mongolia Agricultural University Hohhot China

**Keywords:** AMP‐activated protein kinase, feeding regimens, meat quality, myosin heavy chain isoforms, peroxisome proliferator‐activated receptor‐coactivator‐1α

## Abstract

The effects of two feeding regimens on meat quality, myosin heavy chain (MyHC) types, and key factors regulating muscle fiber type (AMP‐activated protein kinase [AMPK] and peroxisome proliferator‐activated receptor‐coactivator‐1α [PGC‐1α]) in the biceps femoris muscle of Mongolia sheep were investigated. A total of 20 Mongolia sheep were weaning for 90 days and divided into two groups (pasture group (P) and confinement group (C)) at 10.36 ± 0.35 kg of weaning weight. After weaning, sheep were pasture fed or confinement fed for 9 months. The results showed that live weights, carcass weight, intramuscular fat (IMF), and Warner–Bratzler shear force (WBSF) in P group were significantly lower (*p* < .05) than that in C group. Compared with P group, color evaluations with respect to L* and b* values were significantly higher (*p* < .05) in C group. Expression of the MyHC I gene in the P group was significantly higher, while MyHC IIa and MyHC IIb genes expression was significantly lower (*p* < .05) than that in C group. Also, AMPK activity and expression of AMPKα2 and PGC‐1α genes were significantly higher (*p* < .05) in P group compared with C group. The present study indicated that muscle fiber composition was one of the key differences leading to the differences of meat quality in different feeding regimens. AMPK, particularly AMPKα2, and PGC‐1α were considered to be two key factors regulating muscle fiber types in Mongolia sheep. The results support that AMPK activity and the expression of AMPKα2 and PGC‐1α genes may affect the composition of muscle fibers; thus, AMPK activity and the expression of AMPKα2 and PGC‐1α genes had an effect on meat quality by changed composition of muscle fibers.

## Graphical abstract

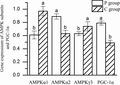

## 1 INTRODUCTION

Muscle fiber was the most basic component unit of muscle quality, which composition and type transformation of muscle fiber would directly affect the meat quality, such as tenderness, flavor, and intramuscular fat (Yin, Sun, Zhang, Bai, & Wang, 2013). Muscle fiber classification method mainly has three kinds: The first method is to simply divided into type I muscle fibers (slow contraction—red muscle fiber—oxidizing muscle fibers) and type II fibers (fast contraction—white muscle fiber—glycolysis muscle fibers). The second type divides muscle fibers into three types according to ATPase activity: type I (slow‐twitch oxidative), type IIA (fast‐twitch oxidative glycolysis), and type IIB (fast‐twitch glycolysis) (Brooke & Kaiser, 1970). The third is based on the myosin heavy chain (MyHC) polymorphism, mammalian skeletal muscle fiber type composition can be divided into four types: MyHC I (slow‐twitch oxidative), MyHC IIa (fast‐twitch oxidative), MyHC IIx (fast‐twitch oxidative‐glycolytic), and MyHC IIb (fast‐twitch glycolytic; Schiaffino & Reggiani, 2011). A shift of muscle fiber from type IIB to type I may cause more tenderness of meat, thus improve meat quality (Choi & Kim, 2009). In recent years, the previous research has reported that meat quality can be improved by altering the composition of muscle fibers (Zhang, Tang, Zhang, Wang, & Wang, 2014). A study in Turkish native sheep breeds found that diameter of type I muscle fibers was positively correlated with tenderness and pH value in longissimus dorsi muscle (LD), and the numbers of type IIA were positively correlated with water holding capacity (WHC) in LD of Akkaraman sheep breeds (Şirin et al., 2017). In another study, when the proportion of type I was high and the proportion of type IIB was low in longissimus dorsi, Korean native cattle had high marbling, more tenderness, and lightness (Hwang, Kim, Jeong, Hur, & Joo, 2010). According to Gil et al. (2003), compared with muscles with lower MyHC I content, muscles with higher MyHC I content showed higher ultimate pH value, but lower dropping loss, and darker surface. These results suggest that muscle fiber types had great influence on meat quality.

Several key gene‐regulated muscle fiber types have been identified, such as AMP‐activated protein kinase (AMPK) and peroxisome proliferator‐activated receptor‐coactivator‐1α (PGC‐1α) (Lin et al., 2002; Röckl et al., 2007). AMPK signaling pathway is central in the regulation of cellular energy, it referred to as cellular energy sensor (Hardie & Sakamoto, 2006). A study had found that chronic administration of 5‐aminoimidazole‐4‐carboxamide‐ribo‐nucleoside (AICAR) which an AMPK agonist can induce muscle fiber types from fast to slow transition (Suwa, Nakano, & Kumagai, 2003). AMPK was also activated during muscle contraction, and regular endurance exercise can induce AMPK activation, accompanied by the conversion of muscle fiber types from fast to slow (Röckl et al., 2007). According to Cantã (2009), AMPK could increase the level of NAD+ in mouse myoblasts, activate Sirtuin (Sirt1), and then regulate Sirt1 downstream gene PGC‐1α. It has been reported that PGC‐1α was involved in conversion and determination of muscle fiber types, lead to the distribution of red/oxidized fiber types increasing (Lin et al., 2002). According to Tetsuo, Takayoshi, Hideaki, Shihori, and Yoriko (2010), the expression of PGC‐1α gene could transform muscle fiber from fast to slow. Overexpression of PGC‐1α in transgenic mice and pigs can promote conversion of glycolytic fibers to oxidative fiber through enhanced mitochondrial respiration and fatty acid oxidation (Zhang et al., 2017).

In recent years, China had introduced many policies prohibiting grazing and restricting grazing. Therefore, the grazing method has gradually changed from natural grazing to house feeding. Many studies over the past few years had reported that feeding regimens can play an important role in animal husbandry, including improved meat quality, growth performance, and fatty acid composition (Carrasco, Panea, Ripoll, Sanz, & Joy, 2009; Fruet et al., 2016; Wang et al., 2018). Feeding regimens also affect the distribution of different types of muscle fibers in farm animals. There are also differences in the distribution of various muscle fibers in the same part of the indoor and the outdoor animals, as the result may be caused by the amount of exercise in the outdoor animals is larger, and the increase in exercise will promote the aerobic metabolism of the muscles and affect the transformation of muscle fibers, thus affecting the distribution of muscle fibers. Exercise can promote the conversion of IIb to IIx to IIa to I muscle fibers, reducing exercise can promote the transformation of muscle fibers in the opposite direction (Gerrard, 2000). According to Petersen, Henckel, Oksbjerg, and Sørensen (1998), the content of IIa in the muscle of the outdoor pigs was higher than that of the indoor pigs, while the contents of IIb and IIx were decreased in outdoor group, in addition, the aerobic metabolism of the muscles of the free‐range pigs was strengthened, and the shape of the meat was affected. In summary, the differences in the distribution and composition of muscle fibers under different feeding regimens may be important reasons for the different quality of meat products.

The aim of the present study was to compare the effect of two feeding regimens, on meat quality, MyHC isoforms, and muscle fiber‐related genes in the biceps femoris muscle of Mongolia sheep.

## 2 MATERIALS AND METHODS

### 2.1 Animals and diets

A total of 20 Mongolia sheep from Inner Mongolia, China were weaning for 90 days and divided into two groups (5 rams and 5 ewes in each group) at 10.36 ± 0.35 kg of weaning weight. After weaning, all sheep were randomly (10 animals and 5 each of rams and ewes/ feeding regimen) according to two different feeding regimens in the subsequent growth phase: pasture feeding regimen (P) and confinement feeding regimen (C). The P group sheep were allowed to graze freely on natural pasture which is semi‐arid grassland for 9 months that mainly consisted of *Stipa gobicao*, *Stipa breviflora*, and *Cleistogenes. Squarrosa,* animals were allowed to move freely in the field of 10^4^ m^2^. While the C group sheep were kept in one pen (16 m^2^) for 9 months in a feedlot and fed a controlled diet of forage consisting of corn stalks, skin of sunflower seed, corn's concentrate and fattening feed.

### 2.2 Samples collection at slaughter

All the animals were sacrificed at 12 months (3 months weaning and 9 months feeding regimes) in a local abattoir (Bayannur Agriculture and Animal Husbandry, Inner Mongolia, China). The live weight was measured before slaughter. After the sheep were slaughtered and fully bloodshot, the head, hooves, internal organs, fur strip, and lymph were removed; after 30 min, the carcass weight was measured. Small portions of *biceps femoris* (BF) were taken from the other part of the carcass within 45 min postmortem were cut into 0.5 cm × 0.5 cm × 1.0 cm pieces, and immediately frozen in liquid nitrogen and stored at −80°C until further analysis.

### 2.3 Meat quality

The meat color of the muscle was evaluated on the muscle surface using a chromatic meter (CR‐410, Konica Minolta, Japan). The average of three measurements was recorded, and the results were expressed as luminance (L*), redness (a*), and yellowness (b*). The initial and ultimate pH were measured on the biceps femoris muscle at 45 min and 24 hr postmortem using a pH meter (pH‐Star; Ingenieurbüro R. Matthäus, Ebenried). The Warner–Bratzler shear force (WBSF, kg/cm^2^) was measured using a tenderness meter (C‐LM3B, Northeast Agricultural University, Harbin, China). Muscle samples (approximately 300 g) were taken from the biceps femoris at 24 hr after slaughter. Then, the samples were heated in a sealed plastic bag in a water bath at 75°C for approximately 45 min, followed by cooling in cold tap water for 40 min. 12 rectangular cores (1 cm^2^), parallel to the longitudinal orientation of the muscle fibers, were taken and analyzed. The greatest and least values for each sample were disregarded, and then, the mean of the remaining values was used to get the final result of WBSF. The meat samples (60 g) were chopped in a commercial mixer‐blender and stirred well and analyzed in triplicate. The protein content was analyzed by Kjeldahl (GB 5009.5‐2016), and intramuscular fat content was analyzed by Soxhlet (GB 5009.6‐2016). The moisture was determined by drying to a constant weight in an oven (105°C), and the ash on the sample residue was measured after drying at 550°C for 12 hr in a muffle furnace.

### 2.4 AMPK activity

Frozen muscle samples (300mg) were homogenized using a polytron homogenizer (XHF‐DY, Scientz) in 5 times their volume of chilled (1–2°C) buffer (wt/vol) consisting of: (0.25 M D‐maimitol (AR, Amresco), 0.05 M Tris–HCl (AR, Amresco, pH 7.4, 4°C), 1 mM EDTA (AR, Sigma), 1 mM DTT (AR, Merck), 1 mM EGTA (AR, Sigma, America), 50 mM NaF (AR, Yongda), and 5 mM sodium pyrophosphate (AR, Yongda). The homogenate was centrifuged at 10,000 r/min for 5 min at 4°C (Underwood et al., 2008). The level of AMPK phosphorylation was used as a control in the analysis of AMPK activity. AMPK activity was measured using a Sheep Phosphorylated Adenosine monophosphate‐activated protein kinase (p‐AMPK) ELISA Assay Kit (Nanjing Jiancheng Bioengineering Institute, Nanjing, China). The kit uses a double‐antibody sandwich enzyme‐linked immuno‐assay (ELISA) to assay the level of p‐AMPK in samples. Enzyme activity was expressed as the level of p‐AMPK (ng/mL).

### 2.5 RNA extraction and RT‐ PCR

Total RNA from the frozen muscle samples was extracted using RNAiso Plus (Takara, Dalian, China) according to the manufacturer's protocol. The concentration and purity of the total RNA was calculated using BioDrop μLite (BioDrop, Cambridge, England). The extracted RNA was digested using gDNA Eraser (Takara). Reverse transcriptions were performed using an M‐MLV reverse transcriptase kit (Takara).

The expression levels of all genes were examined by RT‐PCR using SYBR Premix Ex Taq (Takara). The GAPDH gene was used as the internal reference for evaluation of the AMPKα1, AMPKα2, AMPKγ3, PGC‐1α, MyHC I, MyHC IIa, MyHC IIb, and MyHC IIx genes, because the expression level of GAPDH was basically consistent in each tissue of the animal body (Zhang et al., 2018). The primer sequences used are shown in Table 1. The reaction (25 μl) contained 2 μl pooled cDNA template, 12.5 μl SYBR Premix Ex Taq (Takara), forward and reverse primers (1 μl each), and DNase/RNase‐free water (8.5 μl).The RT‐PCR conditions used for all genes were as follows: 95°C/30 s, 35 cycles of 95°C/5 s, 57°C/30 s and 72°C/30 s, all genes followed by a final extension cycle at 72°C for 10 min. The CT values were quantified using a modified delta‐Ct method and a PCR data analysis program. For all the treated samples, evaluation of 2^−ΔΔ^Ct indicated the fold change in gene expression relative to the control (Livak & Schmittgen, 2001).

**Table 1:** Primer sequences for RT‐PCR

Gene	Primers	Amplicon (bp)	Accession number
AMPKα1	F: TCCGAAGTATTGATGATGA R: ACAGATGAGGTAAGAGAAG	154	XM_004017019.2
AMPKα2	F: ATGAGGTGGTGGAGCAGAGG R: CGTGAGAGAGCCAGAGAGTGAA	131	EU_131097.1
AMPKγ3	F: GTAACCCGTTGAACCCCATT R: CCATCCAATCGGTAGTAGCG	127	EU_477214.1
PGC‐1α	F: TGCTGCTCTGGTTGGTGAAG R: TGAAGGCTCGTTGTTGTACTGATT R:GGAGGAGTCGTGGGAGGAG	166	XM_012179735.2
MyHC I	F: AAGAACCTGCTGCGGCTG R: CCAAGATGTGGCACGGCT	250	XM_012129251.1
MyHC Ila	F: GAGGAACAATCCAATACAAATCTATCT R: CCCATAGCATCAGGACACGA	173	XM_015098655.1
MyHC IIb	F: GACAACTCCTCTCGCTTTGG R: GGACTGTGATCTCCCCTTGA	274	XM_004012706.3
MyHC IIx	F: GGAGGAACAATCCAATGTCAAC R: GTCACTTTTTAGCATTTGGATGAGTTA	178	XM_012114332.2
GAPDH	F: CTCAAGGGCATTCTAGGCTACACT R: GACCATGAGGTCCACCACCCTGT	180	NM_001190390.1

### 2.6 Statistical analysis

Differences in the parameters studied were analyzed using a one‐way analysis of IBM SPSS Statistics 19.0. The LSD test was applied to compare the averages. The average of the standard error media (SEM) was reported. The relationship between the parameters was evaluated by Pearson correlation analysis.

## 3 RESULTS AND DISCUSSION

### 3.1 Meat quality

The results of effects of feeding regimen on Mongolia sheep meat quality are shown in Table 2. The feeding regimens had a significant effect on live weight and carcass weight which were 23.47% and 39.8% higher, respectively, in C group than in P group (*p* < .05). L* and b* values of the P group were 12.45% and 86.77% (*p* < .05) higher than those of C group, respectively, in agree with the study of Kim et al. (2013) that L* and b* values of subcutaneous fat in pasture lambs were higher than those in fed in the closed environment feeding lambs. There was no significant difference in pH value in 45 min and 24 hr postslaughter (*p* > .05). The study showed that the WBSF of P group was significantly lower 14.04% than C group (*p* < .05) indicating that muscle tenderness was better in P group. The IMF content of BF muscle in C group was significantly higher 54.59% than that in P group (*p* < .05), which was consistent with the research results of Angela, Alenka, Silvester, and Drago (2014). Moisture, protein content, ash, and a* values showed no significant difference (*p* > .05) in both groups. It is well known that the composition of the muscle fiber type can effect on the meat quality, such as pH value, flavor, color, tenderness, and so on (Eggert, Depreux, Schinckel, Grant, & Gerrard, 2002; Hwang et al., 2010; Maltin et al., 1998; Żochowska et al., 2005). The differences in meat quality between the two groups in the present study may be explained by the composition of the muscle fibers.

**Table 2:** Effect of feeding regimen on meat quality in Mongolia sheep (*n* = 10)

	P group	C group
Live weight (kg)	37.95 ± 2.25b	46.86 ± 1.72a
Carcass weight(kg)	16.85 ± 1.22b	23.56 ± 2.51a
L*	27.83 ± 2.41a	24.74 ± 2.33b
a*	20.73 ± 1.76a	19.33 ± 3.28a
b*	7.34 ± 1.58a	3.93 ± 1.57b
pH_0_	6.35 ± 0.09a	6.56 ± 0.10a
pH_24_	5.71 ± 0.08a	5.77 ± 0.07a
WBSF (kg/cm^2^)	45.47 ± 6.73b	52.90 ± 5.98a
Intramuscular fat (%)	4.25 ± 0.56b	6.57 ± 0.62a
Moisture (%)	74.11 ± 6.89a	72.21 ± 3.21a
Protein (%)	22.23 ± 1.32a	21.59 ± 1.02a
Ash (%)	1.01 ± 0.06a	1.06 ± 0.09a

Note

Values in rows followed by different letters are significantly different (*p* < .05).

Abbreviation: WBSF, Warner–Bratzler shear force.

### 3.2 Expression of MyHC isoforms genes

**Figure 1:**
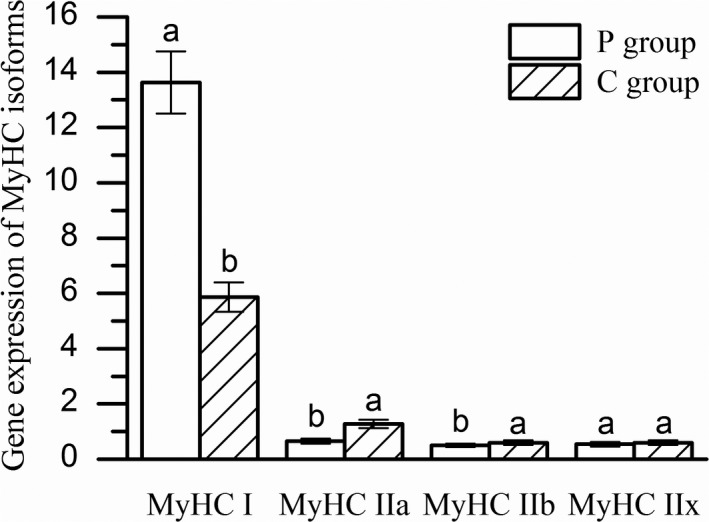
Gene expression of MyHC isoforms in P and C groups (*n* = 10). Columns with different letters for the same isoform are significantly different (*p* < .05)

The effects of feeding regimens on muscle fiber type composition of Mongolia sheep are shown in Figure 1. The expression of MyHC I gene in P group was significantly higher than that in C group (*p* < .05). The expression of the MyHC IIa and MyHC IIb genes in the P group was significantly lower than C group (*p *< .05). Expression of MyHC IIx gene had no significant difference (*p* > .05) in two groups. It is well known that the type of muscle fiber was related to the meat color. Renerre (1990) reported that an increase in the number of type I muscle fibers in skeletal muscle tissue would reduce the color stability of the meat. Pork with higher number and size IIA and IIB muscle fibers, the L* values was increase and WHC (water holding capacity) was decrease (Kim et al., 2013; Larzul et al., 1997). On the contrary, the present study showed that the expression of MyHC IIa and MyHC IIb genes in the C group were higher than P group, while the L* and b* values were lower than the PF group. Therefore, the relationship between meat color and muscle fiber types needs to be further studied in Mongolia sheep. As we all know, MyHC IIb gene had more glycogen than other MyHC isoforms genes (Men, Deng, Xu, & Tao, 2012; Quiroz‐Rothe & Rivero, 2010; Shen et al., 2014). Thus, we expected that the pH values would be lower in C group which had the higher MyHC IIb gene expression in both groups. Contrary to what was expected, there was no statistical significant in PH_0_ and pH_24_ between the two groups in our experiments because fiber proportion and preslaughter glycogen reserve level also affects pH decline (Y M Choi, Ryu, & Kim, 2007; Immonen, Ruusunen, Hissa, & Puolanne, 2000; Ryu & Kim, 2006) The relationship between IMF content and muscle fiber properties is currently controversial (Lefaucheur, 2010). According to Hu, Wang, Zhu, Guo, and Wu (2008), mRNA expression of MyHC I, IIa, and IIx was positively correlated with IMF content in the longissimus dorsi muscle of pigs, and MyHC IIb mRNA expression was opposite. Zhang, Liu, et al. (2014) reported that the MyHC gene may be considered a negative factor with IMF content. In present study, the changes in the gene expression of MyHC IIa and MyHC IIb with IMF content were consistent, but MyHC I was reversed. The proportion of MyHC I fibers was significantly higher in the lowest shear force group in longissimus dorsi muscle of Sutai pigs (Yang, Jie, Chao, & Zhao, 2012). Our results agree in part with previous reports. Therefore, the effects of feeding regimen on meat quality may via altering the composition of muscle fiber types.

It has been demonstrated that exercise had profound effects on the transformation of muscle fiber types. Morifuji, Murakami, and Fujino (2016) suggested that endurance exercise could prevent the conversion of muscle fibers from slow muscle fibers to fast muscle fibers and increase mitochondrial oxidase activity in skeletal muscle of nonobese type 2 diabetic rats. Eight weeks of treadmill training in rats, the results showed that moderate and heavy intensity exercise may lead to transform of skeletal muscle fiber types from fast to slow (Yin et al., 2017). The wild‐type mice were treated with voluntary wheel running for six weeks inducing shift of muscle fiber types from IIb to IIa/x through AMPK mediates adaptive responses to exercise training in skeletal muscle (Röckl et al., 2007). According to Su et al. (2019), endurance exercise lead to convert in MyHC isoforms from oxidative type to the glycolytic type. In present study, the variation of movement in two groups may be one of the main reasons caused the differences of meat quality. The space of the P group was about 1,000 times of the C group. And the P group were allowed to graze freely on natural pasture which semi‐arid grassland so that the pastured sheep had to keep walking for grass. Therefore, the physical activity of the pastured sheep far more than confinement sheep. Results of the present study showed that exercise of Mongolia sheep in different feeding regimen differs and chronic exercise influences the gene expression of MyHC isoforms and further affects meat quality traits. And we could perhaps draw a conclusion that long‐term pasture can lead to the conversion of MyHC IIx, MyHC IIa, and MyHC IIb to MyHC I in the biceps femoris muscle of Mongolia sheep.

### 3.3 Comparison of key factors regulating muscle fiber type of different feeding regimens

**Figure 2:**
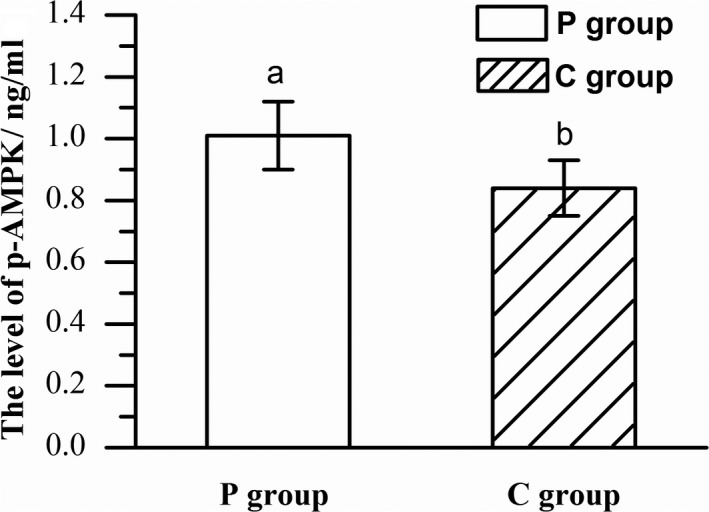
The level of p‐AMPK in P and C groups (*n* = 10). Columns with different letters for the same subunit are significantly different (*p* < .05)

**Figure 3:**
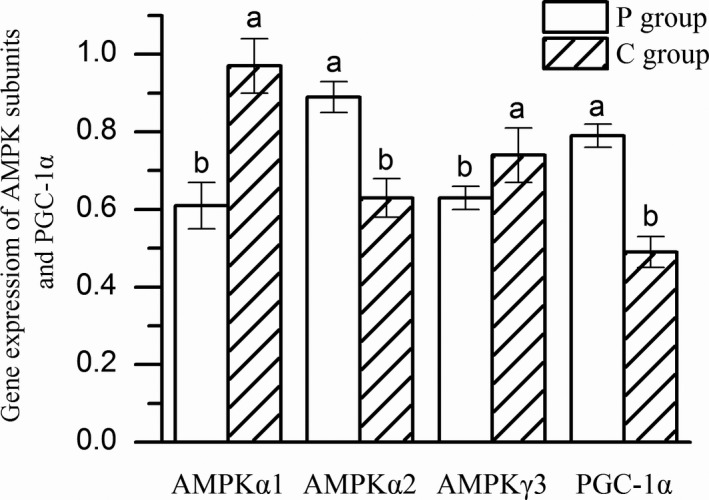
Gene expression of AMPK subunits and PGC‐1α in P and C groups (*n* = 10). Columns with different letters for the same subunit are significantly different

The influence of different feeding regimens on AMPK activity of Mongolia sheep was presented in Figure 2. The level of p‐AMPK in BF muscle of P group was significantly higher than that in C group (*p* < .05). The influence of different feeding regimens on the gene expression of AMPK subunits are shown in Figure 3. Compared with C group, the expression of AMPKα1 and AMPKγ3 genes in BF muscle of P group was significantly lower (*p* < .05), while the expression of the AMPKα2 gene in P group was significantly higher (*p* < .05) than C group. Consistent with the AMPK activity and the expression of AMPKα2 gene, PGC‐1α expression in P group was higher than that in BF muscle of C group (*p* < .05).

AMPK signaling pathway is central in the regulation of cellular energy, it referred to as cellular energy sensor (Hardie & Sakamoto, 2006). According to Ko et al. (2018), aerobic exercise training could increase the activation of AMPK. Energy was consumed during exercise, resulting in a rapid increase in the level of AMP/ATP of the skeletal muscle, and AMPK was activated. Cycling human and mouse wheel movement would cause increased AMPK activity (Zhi‐Ping et al., 2003; Wojtaszewski, Jorgensen, Hellsten, Hardie, & Richter, 2002). Therefore, the increase of AMPK activity in pastured sheep compared with confinement may due to the greater physical exercise before slaughter in P group.

Although skeletal muscle expresses the AMPKα1 and AMPKα2, the AMPKα2 was activated primarily in response to moderate‐intensity endurance exercise in skeletal muscle, promotes skeletal muscle glucose transport, glutamate expression (Nakano et al., 2006). While AMPKα1 was reversed and activated only during tonic muscle contraction (Friedrichsen, Mortensen, Pehmøller, Birk, & Wojtaszewski, 2013), high‐intensity exercise could activate AMPKα2 immediately, but AMPKα1 had no significant change. In vitro experiments further prove that AMPKα2 was more sensitive to ATP than AMPKα1 (Wojtaszewski, Nielsen, Hansen, Richter, & Kiens, 2000). And Mcgee et al. (2003) reported that endurance training could lead to a significant increase in AMPKα2 content. In summary, the activation of different catalytic subunits of AMPK was closely related to exercise intensity, and AMPKα2 was mainly activated in endurance exercise. In present experiments, after a long period of exercise, the pastured sheep may activate the AMPKα2 subunit encoded by the AMPKα2 gene. In agreement with the previous study, AMPKγ3 expression was higher in C group may cause the higher expression of MyHC IIb in C group, since AMPKγ3 gene is a key molecule that regulates the transform to type IIb of muscle fiber (Mahlapuu et al., 2004).

The mouse with a dominant inhibitory mutant of AMPK was intolerant to exercise fatigue, while AMPK activation increases mitochondrial oxidase activity and mitochondrial synthesis (Mu, Jr, Brozinick, Valladares, Bucan, & Birnbaum, 2001), which effect may be mediated by PGC‐1α, as the results of studies transgenic mice expressing a dominant‐negative mutant of AMPK in muscle and knockout mice confirmed the important role of PGC‐1α in the AMPK signaling pathway (Haihong et al., 2002). Overexpression of the PGC1α gene promotes the transformation of glycolytic muscle fibers into the muscle fibers of pigs (Ying, Zhang, Bu, Xiong, & Zuo, 2016). Through the transgenic method, it was found that after transferring PGC‐1α gene into mice, type II muscle fibers showed the characteristics of slow type muscle fibers, and mitochondrial oxidative ability was enhanced, indicating that the specific protein expression level of type I muscle fibers was elevated, and type II muscle fibers could be obtained. It was converted into Ι type muscle fiber (Lin et al., 2002). And Handschin et al. found that in PGC‐1α knockout mice, I and IIa muscle fibers were significantly reduced, and type IIb muscle fibers were significantly elevated (Handschin et al., 2007). Exercise induced activation of PGC‐1α was associated with the activation of AMPK and p38 MAPK (Gibala et al., 2009; Handschin et al., 2007). According to Cantã et al. (2009), AMPK could increase the level of NAD+ in mouse myoblasts, activate Sirtuin (Sirt1), and then regulate Sirt1 downstream gene PGC‐1α. These results indicate that AMPK induced muscle fiber type transition via, at least in part, PGC‐1α gene. As the results of the key factors regulating muscle fiber type in different feeding regimens of Mongolia sheep in present study, the long‐term exercise of pasture sheep could increase the gene expression of AMPKα2, activate AMPK, up‐regulate the gene expression of PGC‐1α, and promote the transformation of muscle fiber type from MyHC IIx, MyHC IIa, and MyHC IIb to MyHC I in the biceps femoris muscle of Mongolia sheep. Our results provide an important basis for further studies of the mechanism of muscle fiber type in different feeding regimens of Mongolia sheep.

## 4 CONCLUSIONS

In present study, pasture sheep showed an advantage over confinement sheep with respect to WBSF of meat quality. And higher AMPK activity and expression of AMPKα2 and PGC‐1α genes caused by pasturing can promote the transformation of muscle fiber types from MyHC IIx, MyHC IIa, and MyHC IIb to MyHC I, which indicated that feeding regimen affect WBSF via alter muscle fiber types by regulating AMPK activity, and expression of AMPKα2 and PGC‐1α genes.

## CONFLICT OF INTEREST

The author states that there is no conflict of interest and financial, personal, or other relations with others or organizations.

## ETHICAL APPROVAL

Animal experiments were approved by the Animal Experiment Committee and conducted in accordance with the "Guide to Animal Experiment System of College of Animal Science, Inner Mongolia Agricultural University." The experiments were carried out in accordance with recommendations made by the European Commission (1997) with the aim of minimizing animal suffering.
